# Measurement, Characterization, and Mapping of COVID-19 Misinformation in Spain: Cross-Sectional Study

**DOI:** 10.2196/69945

**Published:** 2025-06-16

**Authors:** Javier Alvarez-Galvez, Carolina Lagares-Franco, Esther Ortega-Martin, Helena De Sola, Antonio Rojas-García, Paloma Sanz-Marcos, José Almenara-Barrios, Angelos P Kassianos, Ilaria Montagni, María Camacho-García, Maribel Serrano-Macías, Jesús Carretero-Bravo

**Affiliations:** 1 Computational Social Science DataLab (CS2 DataLab) University Institute for Sustainable Social Development (INDESS) University of Cadiz Jerez de la Frontera (Cadiz) Spain; 2 Department of General Economy (Sociology) University of Cadiz Cadiz Spain; 3 Department of Statistic and Operative Research University of Cadiz Cadiz Spain; 4 Department of Behavioural Science Methodology University of Granada Granada Spain; 5 Department of Marketing and Communication University of Cadiz Jerez de la Frontera Spain; 6 Department of Biomedicine, Biotechnology and Public Health University of Cadiz Cadiz Spain; 7 Department of Nursing School of Health Sciences Cyprus University of Technology Limassol Cyprus; 8 Bordeaux Population Health Research Center Université de Bordeaux - Inserm Bordeaux France; 9 Department of Quantitative Methods Universidad Loyola Andalucía Seville Spain

**Keywords:** misinformation, disinformation, conspiracy theories, health beliefs, polarization, vaccine hesitancy, COVID-19, artificial intelligence, AI

## Abstract

**Background:**

The COVID-19 pandemic has been accompanied by an unprecedented infodemic characterized by the widespread dissemination of misinformation. Globally, misinformation about COVID-19 has led to polarized beliefs and behaviors, including vaccine hesitancy, rejection of governmental authorities’ recommendations, and distrust in health institutions. Thus, understanding the prevalence and drivers of misinformation is critical for designing effective and contextualized public health strategies.

**Objective:**

On the basis of a tailored survey on health misinformation, this study aims to assess the prevalence and distribution of COVID-19–related misinformation in Spain; identify population groups based on their beliefs; and explore the social, economic, ideological, and media use factors associated with susceptibility to misinformation.

**Methods:**

A cross-sectional telephone survey was conducted with a nationally representative sample of 2200 individuals in Spain. The study developed the COVID-19 Misinformation Scale to measure beliefs in misinformation. Exploratory factor analysis identified key misinformation topics, and k-means clustering classified participants into 3 groups: convinced, hesitant, and skeptical. Multinomial logistic regression was used to explore associations between misinformation beliefs and demographic, social, and health-related variables.

**Results:**

Three population groups were identified: convinced (1078/2200, 49%), hesitant (666/2200, 30.27%), and skeptical (456/2200, 20.73%). Conspiracy theories, doubts about vaccines, and stories about sudden death emerged as the most endorsed current misinformation topics. Higher susceptibility to misinformation was associated with the female sex, lower socioeconomic status, use of low-quality information sources, higher levels of media sharing, greater religiosity, distrust of institutions, and extreme and unstated political ideologies. Frequent sharing of health information on social networks was also associated with membership in the skeptical group, regardless of whether the information was verified. Interestingly, women were prone to COVID-19 skepticism, a finding that warranted further research to understand the gender-specific factors driving vulnerability to health misinformation. In addition, a geographic distribution of hesitant and skeptical groups was observed that coincides with the so-called empty Spain, areas where political disaffection with the main political parties is greater.

**Conclusions:**

This study highlights the important role of determinants of susceptibility to COVID-19 misinformation that go beyond purely socioeconomic and ideological factors. Although these factors are relevant in explaining the social reproduction of this phenomenon, some determinants are linked to the use of social media (ie, searching and sharing of alternative health information) and probably the political disaffection of citizens who have stopped believing in both the ideologically centrist mainstream parties and the institutions that represent them. Furthermore, by establishing the profile and geographic distribution of the convinced, hesitant, and skeptical groups, our results provide useful insights for public health interventions. Specific strategies should focus on restoring institutional trust, promoting reliable sources of information, and addressing structural drivers of health misinformation linked to gender inequalities.

## Introduction

### Background

The COVID-19 pandemic has not only posed unprecedented challenges to global public health but also has been accompanied by a pervasive infodemic [1,2]. This term, defined by the World Health Organization (WHO) as “an over-abundance of information—some accurate and some not—that makes it hard for people to find trustworthy sources and reliable guidance when they need it” [3], underscores the critical role of information in shaping public responses to social and health emergencies [4]. While access to information is essential, the unprecedented volumes of data surrounding SARS-CoV-2 and COVID-19 has made it increasingly difficult for individuals and institutions to discern scientific evidence from misinformation or disinformation and anecdotal claims [5]. In fact, in this context of global uncertainty, we have been able to observe speculations and conspiracy theories [6] related to different topics, such as the supposed effectiveness of the RNA vaccines, the hidden intentions of global leaders and the pharmaceutical industry, false health treatments (eg, the hydroxychloroquine case), the real origin of the new virus, and even doubts about the existence of the virus [7].

During the recent pandemic, the dissemination of false information about scientific and health-related matters spread faster and more easily than the virus itself [8]. Furthermore, the blurring boundaries between evidence-based knowledge and false or misleading information linked to the new disease introduced substantial complexities to the population’s ability to make informed decisions [5]. Specifically, the dissemination of new health guidelines and the heterogeneous political measures to contain the pandemic through traditional and social media caused an excess of noise around everything related to health (ie, vaccines, treatments, contention measures, etc), which ended up generating social polarization and social divisions that made it difficult to manage and control the health emergency situation [9]. In addition, information overload contributed to cognitive fatigue, which has also been shown to reduce people’s ability to critically evaluate health sources and content [10]. In addition, the role of influential opinion and political leaders has further exacerbated the situation [11], propagating misconceptions and encouraging risky behaviors that may have undermined public health interventions [12].

The COVID-19 infodemic exemplifies how modern information ecosystems, particularly those based on social media communication, can amplify both evidence-based guidance and misinformation at an unprecedented scale [13,14]. These “misinformation ecosystems” have directly influenced social and health behaviors, contributing to vaccine hesitancy [15,16], noncompliance with health measures [17], and the proliferation of pseudoscientific remedies and theories [18], ultimately challenging the effectiveness of governmental and institutional countermeasures [19]. Moreover, as recent studies have shown, scientific content and sources also play a relevant role in the dissemination of misinformation, for example, through the design of poorly elaborated messages via social media platforms or the provision of weak evidence [20]. Therefore, when focusing on the concept of misinformation in health and, specifically, COVID-19, we are faced with a cross-cutting phenomenon that permeates all strata of our societies.

While it is becoming increasingly common to find studies addressing health misinformation and its impact on health systems, it still remains a difficult concept to define due to the fluctuating nature of the social media ecosystem and the diversity of health-related topics it covers, especially when analyzing the subject matter of COVID-19 [21]. To address this complexity, in this study, we adopt a broad definition of health misinformation, encompassing any health-related claim that relies on anecdotal evidence, is false, or is misleading due to the absence of scientific confirmation [22], regardless of whether this information has been issued intentionally or unintentionally [23]. Therefore, our definition incorporates 2 key forms: misinformation, which refers to false information shared without an intent to cause harm [24,25], and disinformation or malinformation, which involves false or partially true information deliberately crafted to deceive or harm specific individuals, social groups, institutions, or nations [26]. Our definition implicitly incorporates the flexibility of the definition by the National Academies of Sciences, Engineering, and Medicine report, which defines science-related misinformation as “information that asserts or implies claims that are inconsistent with the weight of accepted scientific evidence at the time.” This conceptualization underscores the importance of distinguishing between misinformation that directly contradicts well-established evidence and misinformation that may reflect evolving or uncertain scientific consensus. In light of this, our study attempts to capture both types of claims in the COVID-19 context, acknowledging that the epistemic status of some items may be more contentious than others [27].

Although studies have been emerging since the beginning of the COVID-19 pandemic that have addressed misinformation sources, channels, and messages [28-31], in general, less attention has been paid to the exhaustive characterization of the profiles of people who embrace misinformation and particularly understanding which messages have penetrated the audiences. In the literature, cross-sectional studies can be found that have usually used opportunistic samples to understand specific attitudes and behaviors (eg, related to the new vaccines or preventive measures) [32-37], but, to our knowledge, there are no exhaustive and updated studies that allow us to determine the current prevalence of COVID-19 misinformation about the beliefs that may have emerged in recent years and that have progressively taken root in public opinion (eg, doubts about the need for additional vaccines, the origin of the virus, the existence of hidden governmental plans, and sudden deaths, among others). In addition, this highlights the need to provide a comprehensive sociodemographic and contextualized description of these social groups, which could ultimately help us identify the groups that we should target with information campaigns aimed at increasing literacy in COVID-19–related health issues.

In a recent review, political orientation has been found to be a key predictor of misinformation and beliefs in fake news [9]. However, most of the studies reviewed were based in the United States, reflecting a limited geographical focus. To date, this association has not been systematically explored in other contexts, underscoring the need for context-specific research to better understand how political orientation influences susceptibility to misinformation within different sociopolitical frameworks. Particularly in the Spanish case, a study has been carried out analyzing conspiracy beliefs and their association with ideological and religious values based on survey data from the Spanish Foundation for Science and Technology; however, the dataset is not specifically focused on the study of health misinformation related to COVID-19 but on the scientific aspects related to the pandemic [38]. Moreover, although there have been attempts to identify the diversity of misinformation and conspiracy groups through the application of latent profile techniques [39], there is still a lack of comprehensive characterizations of the explanatory factors (demographic, social, economic, political, and media use) of population profiles susceptible to misinformation. In other words, in addition to characterizing the sociodemographic groups of the different profiles that embrace health misinformation, there is a need for studies that provide detailed information on how these groups consume and share information as well as other health aspects that could explain their positioning (eg, general health status, chronic conditions, diagnoses, and COVID-19 vaccination).

### Objectives

Given these knowledge gaps, the aim of this exploratory study was to assess the prevalence and distribution of COVID-19–related misinformation in Spain using data from a representative population sample obtained from the DCODES (Collective dynamics of health opinion contagion: the COVID-19 infodemic and its effects on decision making processes) project. Specifically, we aim to achieve the following specific objectives: (1) identify the issues that generate the greatest social division around the COVID-19 pandemic, (2) classify the main groups around these issues of misinformation about COVID-19, and (3) describe these social profiles by studying the association with a broad set of social determinants that could explain the positioning of these groups.

## Methods

### Design and Setting

This study used a cross-sectional design using telephone surveys conducted from January 2024 to March 2024, targeting individuals aged >18 years residing in Spain. Participants were selected from available databases, with consent obtained before their participation. The telephone survey method was chosen due to its numerous advantages, making it particularly suited to this study’s objectives. First, it allowed for rapid and extensive access to participants, including those in remote or rural areas. Second, it was cost-effective compared to face-to-face interviews, reducing logistical expenses. Third, the standardized administration of questionnaires minimized interviewer-related variability, ensuring greater consistency in data collection. Fourth, it was convenient for respondents, who could complete the survey from their homes, facilitating higher participation rates. Fifth, telephone surveys were particularly effective for addressing sensitive topics, as respondents often felt more secure and perceived greater confidentiality in such interactions. Collectively, these features made telephone surveys an optimal data collection method, ensuring both broad representativeness and logistical feasibility in this study.

Stratified sampling guaranteed representativeness from the different geographic areas of the country based on key demographic factors: age, sex, region, and population size of the areas of residence. Using a multistage sampling approach and drawing on nationally representative datasets, a sampling frame reflecting the demographic composition of Spain was composed. This rigorous methodology minimized selection bias and allowed accurate conclusions to be drawn. The final sample composed of 2200 individuals, with a confidence level of 95% and an estimated error of +2.1 or –2.1 percentage points.

This survey was part of the DCODES project, which aimed to conduct an in-depth analysis of the determinants of misinformation during the COVID-19 pandemic.

### Measures

The questionnaire was developed by a team of 6 researchers with experience in survey methods and misinformation studies through 3 nominal group meetings in which the main contents and variables potentially associated with misinformation on COVID-19 were discussed and consensus was reached on which were the most relevant in the general population. The questionnaire was divided into 4 fundamental blocks of indicators (Table 1).

**Table 1:** Thematic blocks and description of indicators.

Variables	Description of measures
COVID-19 misinformation indicators	The primary set of indicators included the COVID-19 Misinformation Scale, with 12 items related to erroneous, false, or misleading topics that were currently most widely disseminated about COVID-19. These items were extracted from the 3 main platforms for checking hoaxes and fake news in Spain (Newtral, Maldita.es, and EFE Verifica, belonging to the International Fact Checking Network). These indicators were presented on a Likert scale from 1 (totally disagree) to 5 (totally agree) points. The 12 items had high internal consistency (Cronbach α=0.87). The content of each statement is available in the Results section.
Health information use, media sharing, and digital health literacy	The survey incorporated questions about the information medium used the last time to obtain health information and the frequency of online media used to search for health topics. In addition, the survey asked about the frequency with which the individuals shared health topics through social networks and whether they checked this information before sharing it. As another means of measuring the individual’s use and ability to use digital media to obtain health information, we also used the eHealth Literacy Scale [40], an 8-item instrument to measure the individual’s digital health literacy, which has been previously validated in Spain [41].
COVID-19 diagnoses, protective measures, and health status	With the intention of evaluating the association between health and positioning around the different COVID-19 issues, respondents were asked whether they had taken the COVID-19 test, whether they had been vaccinated, and what was the degree of compliance with protective measures during the state of alarm in Spain. This last variable was obtained as the mean of compliance with confinement, social distancing, use of masks, hand washing, and diagnostic testing of contacts. In addition, they were also asked about their self-perceived health and whether they had any chronic disease.
Social and economic indicators	Demographic, social, and economic variables were also incorporated in the questionnaire. Specifically, sex, age, income, size of municipality, educational level, employment status, and nationality were collected. In addition, given the relationship shown with cultural and ideological aspects of the individual and the predisposition to misinformation, we also included political ideology (on a scale of 0-10, where 0 meant extreme left ideology and 10 meant extreme right ideology). Ideology was categorized so that a value of 0 to 1 represented far left wing, a value of 2 to 3 represented left wing, a value of 4 to 6 represented center, a value of 7 to 8 represented right wing, and a value of 9 to 10 represented far right wing. An extra category, nonresponse, for those who preferred not to reveal their ideology was also included. Individual religiosity was also assessed (where 0 meant not religious at all and 10 meant very religious) along with trust in institutions (the Spanish government, political parties, World Health Organization, health personnel, and the Spanish National Health System). Finally, as an analysis of the use of new technologies, we added a question about the knowledge and use of artificial intelligence models, which may pose a risk for increasing misinformation [42], and attitudes toward cryptocurrencies, a relationship that has been found to be associated with conspiracy beliefs [43].

### Data Analyses

First, an exploration of the characteristics of the sample through descriptive statistics was carried out. After that, a dimensionality analysis was conducted on the COVID-19 Misinformation Scale, through an exploratory factor analysis (EFA). To determine the number of initial dimensions of the items, the criteria of the number of eigenvalues >1 and the conceptual meaning of the factors were considered. We used the weighted least squares mean and variance adjusted estimator as a factor extraction method [44] because it is the most suitable method for Likert scale items and the geomin rotation [45] as an oblique rotation method. This rotation method presents the advantage of balancing simplicity and realism when interpreting factor structures. Unlike orthogonal rotations, geomin allows correlations between factors and minimizes small factor loadings, making it particularly suitable for exploring complex latent structures where variables may load on multiple factors, thereby enhancing the interpretability and precision of the results.

Once the factors were derived from the EFA, we aimed to categorize individuals into groups based on their scores on the dimensions, using the k-means clustering technique. In an attempt to offer an exhaustive classification but at the same time a simple and operational one, participants were initially classified into three categories: (1) convinced-—those who believed or followed the majority opinion according to existing research evidence on COVID-19; (2) hesitant—individuals who expressed doubts, characterized by delays in accepting or rejecting COVID-19 information despite its availability; and (3) skeptical—those who tended to believe or follow minority opinion on COVID-19 issues that are erroneous, false, or misleading and commonly based on anecdotal evidence. This decision was made to simplify the results, as the silhouette method indicated that the 3-cluster solution provided a better classification than other groupings. Accordingly, these 3 categories were used to obtain the different individual profiles by testing their relationship with the variables associated with health information resources and use, COVID-19 diagnoses, health status, sociodemographic variables, ideology, and knowledge of technologies through a multinomial logistic regression model. The group of those individuals who were considered convinced was considered as the reference category, and independent variables with n categories were recategorized into n–1 dichotomous variables.

All the analyses were conducted using R (version 4.1.2; R Foundation for Statistical Computing) and RStudio (Posit PBC). To perform the EFA analyses, the *Lavaan* package [46] was used, which incorporates the most up-to-date estimators for categorical variables.

### Ethical Considerations

This study was approved by the Ethics Committee on Non-Biomedical Experimentation and Genetically Modified Organisms (CEENB-GMOs) of the University of Cadiz (005_2024). All data collected through the telephone survey were fully anonymized prior to analysis. No personally identifiable information was recorded or stored, ensuring complete confidentiality of participants and no financial or material compensation was provided to participants. Participation in the study was voluntary, and verbal informed consent was obtained at the beginning of the interview process.

## Results

Table 2 shows the characteristics of the population of the representative sample of 2200 people that participated in this study. As can be seen, the sample was clearly balanced in accordance with the sex and age of the interviewees. In terms of the variables associated with COVID-19, a substantial percentage of vaccination (2088/2200, 94.91%) and a high follow-up of the measures during the pandemic (mean 4.47 out of 5, SD 0.69) were found. Almost 39.05% (859/2200) of the population had been diagnosed with COVID-19 at the time of the survey.

**Table 2:** Summary of the sample characteristics by sex (N=2200).

Characteristics		Overall	Male (n=1060)	Female (n=1140)
**Age group (y), n (%)**				
	18-34	478 (21.73)	238 (22.45)	240 (21.05)
	35-49	624 (28.36)	314 (29.62)	310 (27.19)
	50-64	574 (26.09)	282 (26.6)	292 (25.61)
	>64	524 (23.82)	226 (21.32)	298 (26.14)
**Income level (€; US $1=€0.9267), n (%)**				
	<900	181 (8.23)	73 (6.89)	108 (9.47)
	901-1200	230 (10.45)	85 (8.02)	145 (12.72)
	1201-1800	334 (15.18)	150 (14.15)	184 (16.14)
	1801-2400	332 (15.09)	161 (15.19)	171 (15)
	2401-3000	327 (14.86)	198 (18.68)	129 (11.32)
	3001-4500	292 (13.27)	161 (15.19)	131 (11.49)
	>4500	177 (8.05)	104 (9.81)	73 (6.4)
	N/A^a^	327 (14.86)	128 (12.08)	199 (17.46)
**Size of the municipality (inhabitants), n (%)**				
	<10,000	467 (21.23)	228 (21.51)	239 (20.96)
	10,001-50,000	563 (25.59)	263 (24.81)	300 (26.32)
	50,001-100,000	227 (10.32)	116 (10.94)	111 (9.74)
	100,001-400,000	470 (21.36)	203 (19.15)	267 (23.42)
	>400,000	473 (21.5)	250 (23.58)	223 (19.56)
**Education level, n (%)**				
	Basic or primary	253 (11.59)	112 (10.63)	141 (12.49)
	Professional training or bachelor’s degree	892 (40.86)	466 (44.21)	426 (37.73)
	University degree	798 (36.56)	363 (34.44)	435 (38.53)
	Postgraduate	240 (10.99)	113 (10.72)	127 (11.25)
**Work status, n (%)**				
	Working	1264 (57.45)	664 (62.64)	600 (52.63)
	Unemployed	536 (24.36)	258 (24.34)	278 (24.39)
	Retired	203 (9.23)	76 (7.17)	127 (11.14)
	Student	88 (4)	49 (4.62)	39 (3.42)
	Unpaid work at home	73 (3.32)	1 (0.09)	72 (6.32)
	N/A	36 (1.64)	12 (1.13)	24 (2.11)
**Nationality, n (%)**				
	Spanish	2054 (93.36)	991 (93.49)	1063 (93.25)
	Other (not Spanish)	146 (6.67)	69 (6.51)	77 (6.75)
**Last source of health information** **, n (%)**				
	Internet	486 (22.09)	233 (21.98)	253 (22.19)
	Family or friends	275 (12.5)	134 (12.64)	141 (12.37)
	Books and newspapers	83 (3.77)	43 (4.06)	40 (3.51)
	Health staff	1332 (60.55)	639 (60.28)	693 (60.79)
	N/A	24 (1.09)	11 (1.04)	13 (1.14)
**Online resources for health information, n (%)**				
	Social media	211 (9.59)	90 (8.49)	121 (10.61)
	Online digital media	244 (11.09)	131 (12.36)	113 (9.91)
	Wikipedia	343 (15.59)	148 (13.96)	195 (17.11)
	Institutional webs	522 (23.73)	248 (23.4)	274 (24.04)
	Google	375 (17.05)	170 (16.04)	205 (17.98)
	Did not use online media	424 (19.27)	226 (21.32)	198 (17.37)
	N/A	81 (3.68)	47 (4.43)	34 (2.98)
**Sharing health information on social media, n (%)**				
	Never	1563 (71.05)	769 (72.55)	794 (69.65)
	1 day a month	312 (14.18)	148 (13.96)	164 (14.39)
	1 day a week	198 (9)	78 (7.36)	120 (10.53)
	≥2 days a week	125 (5.68)	63 (5.94)	62 (5.44)
	N/A	2 (0.09)	2 (0.19)	0 (0)
**Evaluation of information shared, n (%)**				
	Yes	698 (34.23)	328 (33.74)	370 (34.68)
	No	1502 (65.77)	732 (66.26)	770 (65.32)
**Self-perceived health, n (%)**				
	Bad or fair	280 (12.73)	152 (14.34)	128 (11.23)
	Good	1327 (60.32)	665 (62.74)	662 (58.07)
	Very good	516 (23.45)	214 (20.19)	302 (26.49)
	Excellent	77 (3.5)	29 (2.74)	48 (4.21)
**Chronic conditions, n (%)**				
	Yes	1433 (65.14)	737 (69.53)	696 (61.05)
	No	767 (34.86)	323 (30.47)	444 (38.95)
**COVID-19 diagnoses, n (%)**				
	Yes	859 (39.05)	420 (39.62)	439 (38.51)
	No	1341 (60.95)	640 (60.38)	701 (61.49)
**COVID-19 vaccination, n (%)**				
	Yes	2088 (94.91)	1000 (94.34)	1088 (95.44)
	No	112 (5.09)	60 (5.66)	52 (4.56)
**AI^b^ knowledge, n (%)**				
	I do not know anything about it	245 (11.14)	97 (9.15)	148 (12.98)
	I have heard about it, but I do not know much about it	1085 (49.32)	477 (45)	608 (53.33)
	I know AI, but I do not use it	410 (18.64)	217 (20.47)	193 (16.93)
	I know AI and I use it	452 (20.55)	266 (25.09)	186 (16.32)
	N/A	8 (0.36)	3 (0.28)	5 (0.44)
**Cryptocurrency knowledge, n (%)**				
	I do not know anything about it	446 (20.27)	173 (16.32)	273 (23.95)
	I have heard about it, but I do not know much about it	1159 (52.68)	504 (47.55)	655 (57.46)
	I know cryptocurrencies, but I do not invest	457 (20.77)	286 (26.98)	171 (15)
	I know cryptocurrencies and I invest in them	129 (5.86)	95 (8.96)	34 (2.98)
	N/A	9 (0.41)	2 (0.19)	7 (0.61)
**Political ideology, n (%)**				
	Far left wing	165 (7.5)	73 (6.89)	92 (8.07)
	Left wing	418 (19)	195 (18.4)	223 (19.56)
	Center	1,085 (49.32)	561 (52.92)	524 (45.96)
	Right wing	211 (9.59)	103 (9.72)	108 (9.47)
	Far right wing	111 (5.05)	57 (5.38)	54 (4.74)
	No response	210 (9.55)	71 (6.7)	139 (12.19)
**No trust in institutions, n (%)**				
	Spanish government	1077 (48.95)	533 (50.28)	544 (47.72)
	Political parties	1613 (73.32)	794 (74.91)	819 (71.84)
	World Health Organization	432 (19.64)	243 (22.92)	189 (16.58)
	Health staff	68 (3.09)	34 (3.21)	34 (2.98)
	Spanish National Health System	230 (10.45)	109 (10.28)	121 (10.61)
COVID-19 compliance measures (Gaussian), mean (95% CI)		4.47 (4.44-4.50)	4.33 (4.28-4.38)	5.00 (4.40-5.00)
Health online literacy (Gaussian), mean (95% CI)		3.29 (2.38-4.00)	3.25 (2.38-3.88)	3.38 (2.50-4.00)
Degree of religiosity (Gaussian), mean (95% CI)		3.85 (3.78-3.91)	3.58 (3.41-3.79)	4.09 (3.90-4.28)

^a^N/A: not available due to missing data.

^b^AI: artificial intelligence.

Regarding the variables that could be associated with beliefs, attitudes, or knowledge that led to misinformation, there was a high level of distrust in political parties (1613/2200, 73.32%) and in the Spanish government (1077/2200, 48.95%). The general population had a medium level of digital health literacy (3.29 out of 5), with the value being somewhat higher in women. The main source of obtaining information on health was from health care professionals (1332/2200, 61.21%), while online information was obtained from institutional websites (522/2200, 24.63%), Google (375/2200, 17.7%), and Wikipedia (343/2200, 16.19%). Up to 34.23% (698/2200) of the participants stated that they did not check the health information they shared through online media.

**Figure 1:**
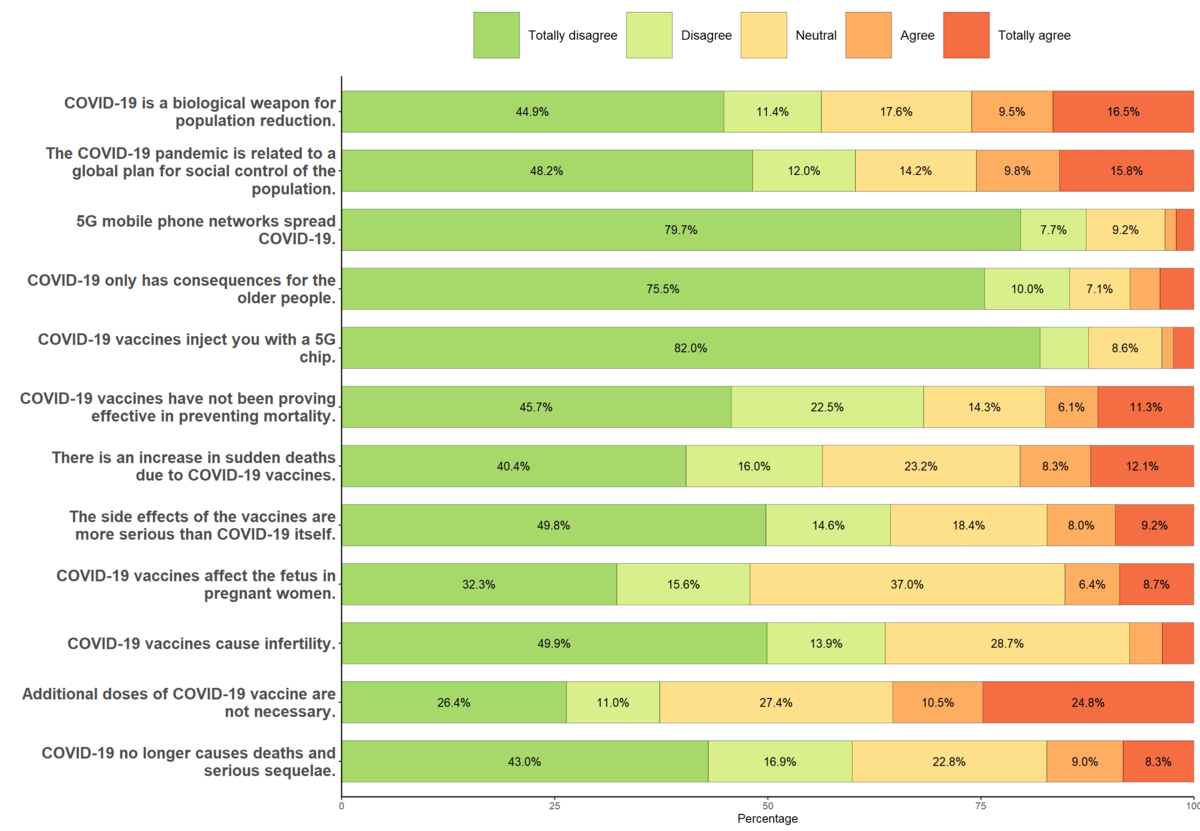
Summary of the COVID-19 misinformation statements.

Figure 1 shows the results of the COVID-19 Misinformation Scale. The items on the need for new doses of vaccines (777/2200, 35.32% thought there is no need for more) and the conspiracy surrounding COVID-19 (572/2200, 26% thought it was a biological weapon, and 559/2200, 25.41% thought it was related to a population control plan) stood out. They also negatively highlighted the sudden increase in deaths (862/2200, 39.18% strongly agreed, agreed or neutral). By contrast, the items concerning the beliefs about the relationship between COVID-19 and 5G mobile network were the ones that caused the least doubts in people, together with the statement that this disease only affected older people.

To summarize the information and investigate the structure of these 12 items, a factor analysis was performed. The Kaiser-Meyer-Olkin statistic and Bartlett test showed that the data were suitable for factor analysis (Kaiser-Meyer-Olkin=0.91; *P*<.001). The results are presented in Table 3. As there were 3 eigenvalues >1 in the polychoric correlation matrix, factorial solutions from 2 to 5 dimensions were reviewed; it was found that the one that made the most conceptual sense was the one with 4 factors, with an explained variance of 64.8%. After performing the geomin rotation, all 12 items presented factor loadings >0.4 without cross loadings between factors, and only 1 item (ie, COVID-19 no longer caused sequelae) had a communality <0.3, although it remained in the model because it had a factor loading >0.4.

**Table 3:** Factorial structure of the COVID-19 Misinformation Scale.

COVID-19 Misinformation Scale items	Factor 1: global plan	Factor 2: health beliefs	Factor 3: vaccine hesitancy	Factor 4: fertility impact	*R* ^2^
COVID-19 is a biological weapon for population reduction.	*0.94* ^a^	–0.011	0.001	0.009	0.883
The COVID-19 pandemic is related to a global plan for social control of the population.	*0.916*	0.026	0.058	–0.002	0.954
5G mobile phone networks spread COVID-19.	0.355	*0.654*	–0.097	0.001	0.703
COVID-19 only has consequences for the older people.	0.001	*0.849*	0.002	–0.235	0.515
COVID-19 vaccines inject you with a 5G chip.	0.182	*0.686*	0.037	0.024	0.726
COVID-19 no longer causes deaths and serious sequelae.	–0.002	*0.442*	0.263	–0.256	0.241
COVID-19 vaccines have not been shown to be effective in preventing mortality.	0.08	–0.018	*0.615*	–0.064	0.388
There is an increase in sudden deaths due to COVID-19 vaccines.	0.021	–0.029	*0.748*	0.086	0.653
The side effects of the vaccines are more serious than COVID-19 itself.	–0.11	0.031	*0.969*	0.015	0.842
Additional doses of COVID-19 vaccine are not necessary.	0.037	0.021	*0.553*	–0.011	0.347
COVID-19 vaccines affect the fetus in pregnant women.	0.062	–0.004	0.007	*0.843*	0.779
COVID-19 vaccines cause infertility.	–0.003	0.106	0.227	*0.61*	0.745
Eigenvalues	6.443	1.101	1.003	0.878	—^b^
Variance explained (%)	20.6	38.6	55	64.8	—

^a^Italic indicate the highest factor loading for each item.

^b^Not applicable.

Factor 1 grouped the 2 items associated with the COVID-19 conspiracy ideas of the existence of a global plan to reduce or control the population. The second factor grouped general health beliefs, such as the link between COVID-19 and 5G technologies, and the supposed impact of the new disease on older groups and mortality. The third factor grouped the claims associated with vaccines, while the fourth factor grouped the 2 items on the fertility consequences of the pandemic. A k-means cluster analysis was used to identify distinct groups of individuals based on their beliefs about the 4 factors extracted from the EFA. The aim of this analysis was to detect 3 distinct subgroups within the population, those who were informed of certain information about COVID-19 and those who had doubts and were skeptics (people who tended to agree with certain COVID-19 misinformation topics). The mean punctuation of the factors by groups can be seen in Table 4.

**Table 4:** Distribution of 3 clusters of individuals (k-means clustering; N=2200).

Social profiles	Factor 1: global plan, mean (SD)	Factor 2: health beliefs, mean (SD)	Factor 3: vaccine hesitancy, mean (SD)	Factor 4: fertility impact, mean (SD)	Individuals, n (%)
Convinced	1.13 (0.33)	1.30 (0.39)	1.71 (0.61)	1.61 (0.72)	1078 (49)
Hesitant	2.93 (0.88)	1.65 (0.57)	2.57 (0.75)	2.33 (0.85)	666 (30.27)
Skeptical	4.47 (0.70)	2.33 (0.91)	3.77 (0.83)	3.43 (0.96)	456 (20.73)

The largest group was composed of what we have called convinced individuals, accounting for almost half of the sample (1078/2200, 49%), while 30.27% (666/2200) of the individuals were classified as hesitant (ie, individuals reluctant to believe COVID-19–related information), and 20.73% (456/2200) of the individuals were in the skeptical group. Within the latter group, the score on the factor about conspiracies stood out, with a mean close to the maximum of 5 as well as a score that was also high on the statements about vaccines. The 2 factors, vaccines and conspiracies, were the main factors to classify the people who believed misinformation about COVID-19 (the factor with the biggest mean score in the hesitant and skeptical individuals).

The final step involved determining the variables associated with membership in these groups through a multinomial regression analysis, the results of which are presented in Table 5. With these variables, the regression model was able to detect 64.82% (1426/2200) of the people in their corresponding group. Among the socioeconomic variables, sex stood out, with a higher probability of women belonging to the skeptical group (OR 1.699; 95% CI (1.187-2.433); *P*=.004). In addition, both the education and income level variables acted in a similar way—the higher the level of education and income, the lower the probability of belonging to the skeptical group, adding also, in the case of educational level, a lower probability of belonging to the hesitant group. The size of the municipality also influenced the results with people in intermediate-sized municipalities (50,000-100,000 inhabitants) having a lower probability of belonging to the group with more skeptics (ie, the skeptical group).

**Table 5:** Multinomial logistic regression with group of misinformation as the dependent variable.

Convinced (reference)				Hesitant					Skeptical		
Category				OR^a^ (95% CI)		*P* value		OR (95% CI)			*P* value
**Sex**											
	Men			1		—^b^		1			—
	Women			1.254 (0.953-1.65)		.12		1.699 (1.187-2.433)			.004^c^
**Age group (y)**											
	18-34			1		—		1			—
	35-49			1.014 (0.698-1.474)		.94		0.88 (0.546-1.416)			.60
	50-64			0.661 (0.433-1.009)		.06		0.729 (0.43-1.237)			.24
	>64			0.591 (0.317-1.101)		.098		0.578 (0.252-1.325)			.12
**Income level (€; US $1=€0.9267)**											
	<900			1		—		1			—
	901-1200			1.005 (0.561-1.801)		.99		0.628 (0.333-1.186)			.15
	1201-1800			1.24 (0.723-2.129)		.43		0.415 (0.222-0.775)			.006^c^
	1801-2400			0.936 (0.536-1.635)		.82		0.288 (0.149-0.56)			<.001^d^
	2401-3000			0.793 (0.448-1.405)		.43		0.321 (0.164-0.629)			.001^c^
	3001-4500			0.83 (0.461-1.495)		.54		0.237 (0.115-0.491)			<.001^d^
	>4500			0.947 (0.494-1.814)		.87		0.317 (0.138-0.726)			.007^c^
**Size of the municipality (inhabitants)**											
	<10,000			1		—		1			—
	10,001-50,000			1.257 (0.866-1.824)		.23		1.227 (0.775-1.941)			.38
	50,001-100,000			0.692 (0.426-1.124)		.14		0.456 (0.235-0.884)			.02^e^
	100,001-400,000			0.993 (0.671-1.469)		.97		0.783 (0.473-1.295)			.34
	>400,000			1.31 (0.876-1.958)		.19		0.955 (0.565-1.614)			.86
**Education level**											
	Basic or primary			1		—		1			—
	Professional training or bachelor’s degree			0.589 (0.367-0.947)		.03^e^		0.481 (0.278-0.833)			.009^c^
	University degree			0.453 (0.273-0.751)		.002^c^		0.272 (0.148-0.503)			<.001^d^
	Postgraduate			0.405 (0.214-0.769)		.006^c^		0.122 (0.047-0.318)			<.001^d^
**Work status**											
	Working			1		—		1			—
	Unemployed			0.981 (0.577-1.667)		.94		0.626 (0.306-1.279)			.199
	Retired			1.216 (0.751-1.971)		.43		0.791 (0.44-1.42)			.43
	Student			1.235 (0.647-2.355)		.52		0.553 (0.213-1.436)			.22
	Unpaid work at home			0.697 (0.329-1.478)		.35		0.413 (0.146-1.171)			.10
**Nationality**											
	Spanish			1		—		1			—
	Other (not Spanish)			1.437 (0.841-2.455)		.18		1.471 (0.774-2.795)			.24
**Last source of health information**											
	Internet			1		—		1			—
	Family or friends			0.589 (0.375-0.925)		.022^e^		0.659 (0.383-1.133)			.13
	Books and newspapers			0.446 (0.211-0.943)		.04^e^		0.279 (0.097-0.8)			.02^e^
	Health staff			0.705 (0.507-0.979)		.04^e^		0.452 (0.297-0.686)			<.001^d^
**Online resources for health information**											
	Social media			1		—		1			—
	Online digital media			0.884 (0.518-1.511)		.65		0.978 (0.496-1.927)			.95
	Wikipedia			0.833 (0.503-1.381)		.48		1.223 (0.657-2.277)			.53
	Institutional webs			0.641 (0.399-1.03)		.06		0.453 (0.24-0.856)			.02^e^
	Google			0.753 (0.45-1.258)		.28		0.841 (0.443-1.597)			.60
	Did not use online media			0.876 (0.498-1.542)		.65		1.258 (0.617-2.566)			.53
**Sharing health information on social media**											
	Never			1		—		1			—
	1 day a month			1.241 (0.872-1.767)		.23		1.313 (0.814-2.115)			.26
	1 day a week			2.691 (1.745-4.149)		<.001^d^		2.498 (1.417-4.404)			.002^c^
	≥2 days a week			1.475 (0.803-2.709)		.21		2.43 (1.195-4.942)			.01^e^
**Contrast the information shared**											
	Yes			1		—		1			—
	No			1.121 (0.846-1.485)		.43		0.836 (0.576, 1.215)			.35
**Self-perceived health**											
	Bad or fair			1		—		1			—
	Good			1.369 (0.9-2.084)		.14		0.929 (0.56-1.542)			.78
	Very good			1.468 (0.903-2.388)		.12		1.145 (0.636-2.061)			.65
	Excellent			1.427 (0.618-3.293)		.41		0.601 (0.194-1.86)			.38
**Chronic conditions**											
	Yes			1		—		1			—
	No			0.777 (0.581-1.04)		.09		1.169 (0.788-1.734)			.44
**COVID-19 diagnoses**											
	Yes			1		—		1			—
	No			1.095 (0.838-1.431)		.51		0.968 (0.681-1.375)			.86
**COVID-19 vaccination**											
	Yes			1		—		1			—
	No			2.489 (1.175-5.272)		.02^e^		7.649 (3.54-16.524)			<.001^d^
**AI^f^ knowledge**											
	I do not know anything about it			1		—		1			—
	I have heard about it, but I do not know much about it			0.998 (0.598-1.667)		.99		0.62 (0.341-1.128)			.12
	I know AI, but I do not use it			1.025 (0.562-1.869)		.94		0.77 (0.37-1.604)			.49
	I know AI, and I use it			0.595 (0.324-1.091)		.09		0.492 (0.236-1.027)			.06
**Cryptocurrency knowledge**											
	I do not know anything about it			1		—		1			—
	I have heard about cryptocurrencies, but I do not know much about it			0.96 (0.658-1.402)		.83		0.731 (0.448-1.19)			.21
	I know cryptocurrencies, but I do not invest			0.815 (0.509-1.306)		.40		0.688 (0.374-1.265)			.22
	I know cryptocurrencies, and I invest in them			1.115 (0.594-2.094)		.74		1.035 (0.468-2.29)			.93
**Political ideology**											
	Far left wing			1		—		1			—
	Left wing			0.999 (0.574-1.74)		.99		1.578 (0.711-3.502)			.26
	Center			1.323 (0.797-2.198)		.28		1.695 (0.818-3.511)			.16
	Right wing			1.682 (0.905-3.126)		.10		1.175 (0.486-2.843)			.72
	Far right wing			1.118 (0.505-2.473)		.78		3.138 (1.229-8.013)			.02^e^
	No response			2.366 (1.14-4.911)		.02^e^		3.273 (1.289-8.309)			.01^e^
**Trust in institutions**											
	**Spanish government**										
		Yes	1		—		1			—	
		No	1.359 (0.983-1.88)		.06		1.198 (0.753-1.906)			.45	
	**Political parties**										
		Yes	1		—		1			—	
		No	1.753 (1.298-2.369)		<.001^d^		2.271 (1.499-3.441)			<.001^d^	
	**World Health Organization**										
		Yes	1		—		1			—	
		No	1.715 (1.176-2.501)		.005^c^		3.612 (2.335-5.588)			<.001^d^	
	**Health staff**										
		Yes	1		—		1			—	
		No	1.249 (0.379-4.116)		.71		2.176 (0.658-7.201)			.20	
	**Spanish National Health System**										
		Yes	1		—		1			—	
		No	1.898 (1.076-3.347)		.03^e^		4.441 (2.446-8.062)			<.001^d^	
COVID-19 compliance measures (continuous)				0.776 (0.626-0.961)		.02^e^		0.673 (0.524-0.866)			.002^c^
Health online literacy (continuous)				0.986 (0.849-1.144)		.85		1.166 (0.965-1.409)			.11
Degree of religiosity (continuous)				1.145 (1.094-1.197)		<.001^d^		1.206 (1.138-1.279)			<.001^d^

^a^OR: odds ratio.

^b^Not available.

^c^Statistically significant at the level of <.01.

^d^Statistically significant at the level of <.001.

^e^Statistically significant at the level of <.05.

^f^AI: artificial intelligence.

Regarding the time of information and the search for health resources, it was observed that those who sought information through health personnel and written media (ie, books and newspapers) were less likely to belong to the skeptical group than those who searched on the internet. Similarly, among those who searched on the internet, those who searched on institutional websites were more likely to belong to the convinced group. Sharing on social media more frequently was associated with a greater probability of belonging to the skeptical group; however, there was no relationship with contrasting the information before sharing it. The degree of digital health literacy of the individual, as measured by the eHealth Literacy Scale, also showed no relationship with the group to which they belonged.

With respect to certain individual thoughts, we found some relationships of interest. Political ideology showed how individuals on the far right and even those who did not want to declare their ideology (ie, possibly those showing political disaffection) were more likely to belong to the skeptical and hesitant groups with respect to individuals who held a moderate political position (whether they positioned themselves on the left or on the right on the ideological scale). In turn, distrust in certain institutions was shown to be a factor related to skepticism (specifically, distrust in political parties, the WHO, and the Spanish National Health System). The same relationship was shown for religiosity—the higher the degree of religiosity, the greater the probability of belonging to the skeptical group.

Reviewing the variables associated with the COVID-19 pandemic and health, not having been vaccinated for COVID-19 and lower compliance with protective measures during the pandemic were associated with individuals belonging to the skeptical group. By contrast, diagnosis of COVID-19, having chronic conditions, and self-perceived health did not present statistically significant relationships.

**Figure 2:**
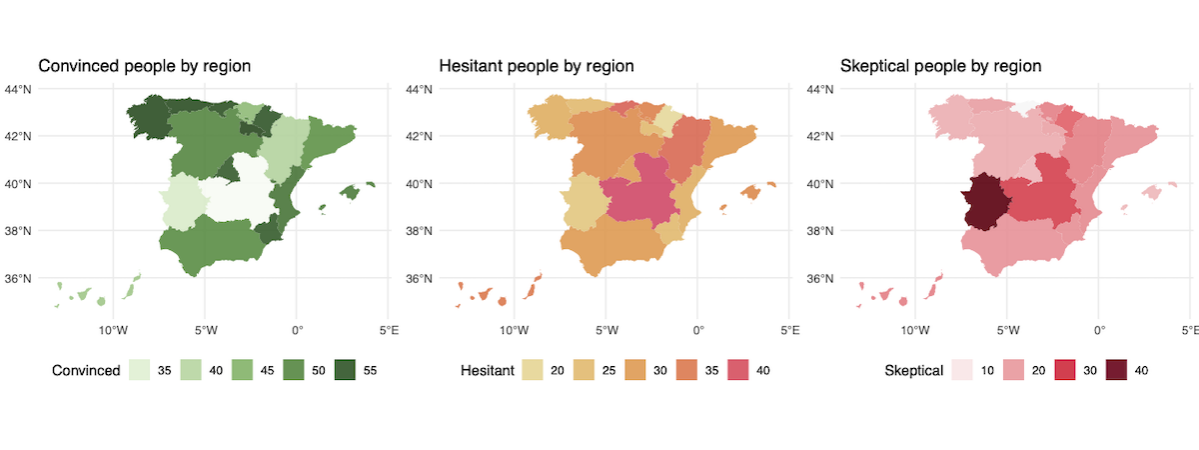
Geographic distribution of convinced, hesitant, and skeptical groups.

Finally, to obtain a contextualized representation of the different groups that we had characterized, we proceeded to study their regional distribution in the country as a whole. Figure 2 shows the maps for the 3 groups: convinced, hesitant, and skeptical. The spatial distribution of these groups on the map of Spain showed that the skeptical groups were found to a greater extent in the so-called empty Spain (particularly in regions such as Extremadura and Castilla-La Mancha), that is, those rural areas of the country characterized by a low population density due to the exodus of young people to the main urban areas. This is a trend that to some extent was also observed with the individuals in the hesitant group, who were also more prevalent among regions of lower population density.

## Discussion

### Principal Findings

This study sheds light on how misinformation related to COVID-19 spread through different social strata of the Spanish population. In line with previous research conducted in other countries, this study reinforces the assumption that social factors, such as political ideology, socioeconomic status, and trust in institutions, play a significant role in determining susceptibility to misinformation [47,48]. However, unlike other studies, our research provides a geographic identification and a comprehensive characterization of the social profiles that are at risk of using erroneous, false, or misleading information as well as a detailed description of the pandemic-related topics that, even today, continue to generate social polarization among the population. Specifically, we have detected that the social profiles most susceptible to misinformation in Spain—in addition to belonging to extreme right-wing ideologies, presenting a low level of socioeconomic status and having a greater distrust of institutions and public health systems—tend to a greater extent to be women without a defined political orientation (possibly due to political disaffection), who use lower-quality information sources (such as social media) and generally unofficial ones, even though they easily share the health information they find through these unofficial media. Likewise, they are groups that reject the COVID-19 vaccines and have shown a lower follow-up of the protective measures decreed by the health authorities during the pandemic.

Among the most relevant topics on misinformation related to COVID-19, it can be emphasized that the themes of the ineffectiveness of additional vaccine doses, pandemic conspiracy theories, and claims of a sudden rise in mortality rates were the topics that elicited the most doubt or agreement within the Spanish population. More than 20% of respondents believed that COVID-19 vaccines lead to a sudden rise in mortality, a perception that likely undermines public motivation to receive further doses. Although Spain is a country with high vaccination rates, these data show that it is necessary to improve the quality of the information provided to the population about the beneficial effects of vaccination and, in particular, existing evidence related to COVID-19 vaccination data. By contrast, the items on the relationship between COVID-19 and 5G mobile phone network are the ones that cause the least doubts in people, as in other studies that have shown that the narrative linking the onset of the pandemic to 5G antennas is the content that people were least likely to believe [30]. In any case, it is necessary to take into account that a small percentage of the population believes this type of hoax.

Cluster analysis allowed us to obtain 3 groups of individuals based on their beliefs toward COVID-19 content. Being a study with a representative sample of the Spanish population, it is relevant to mention that 20.73% (456/2200) of the respondents have been classified in the group of skeptical individuals or the cluster that tends to believe erroneous, false, or misleading information (ie, commonly based on anecdotal evidence more than on real facts and existing scientific evidence). In addition, 30.27% (666/2200) of the participants could be classified as hesitant individuals who express doubts about the current evidence regarding COVID-19. This percentage is notably high for a general population study and reflects a significant level of uncertainty around COVID-19 among the Spanish population. According to this finding, a work conducted by the Spanish Foundation for Science and Technology in 2022 on scientific misinformation in Spain found that more than a quarter of the population living there received false or misleading information about science on a weekly basis [49]. These data, combined with our findings, underscore the magnitude of the problem of misinformation and the risk of having a high percentage of hesitant population (ie, those who are more susceptible to behavioral changes based on the influence of external information sources independent of their quality). Furthermore, it becomes clear that misinformation not only affects all social strata but also has a clear impact on public health through health behaviors (eg, the rejection of vaccines and protective measures against COVID-19).

The factors related to COVID-19 that best differentiated the groups were those associated with conspiracy theories and COVID-19 vaccination. This finding aligns with previous research [50], demonstrating a strong link between belief in misinformation and a conspiratorial worldview, where individuals suspect that both government and institutions are the root of societal problems. Besides, the strength of content related to antivaccine movements stands out in our study, which is positioned as one of the most relevant misinformation topics in other studies [23], and it is one of the most relevant topics among COVID-19 skeptics who embrace misinformation and conspiracy theories. From these findings, it is evident that the hoaxes associated with vaccines are one of the most difficult narratives for institutions to alleviate [35].

In addition to the analyzed data on the prevalence of misinformation in Spain and its social determinants, our study has identified an interesting geographic distribution of misinformed groups that curiously coincides with the rural areas belonging to the so-called empty Spain, regions that, despite being led by both right-wing and left-wing political parties (eg, Castilla-La Mancha and Extremadura, respectively), have the common characteristic of having a low density of population compared with other country areas. Thus, the higher prevalence of the skeptical group in these areas could be due to the high degree of rurality that could imply, on average, both a lower socioeconomic status of the population (ie, lower education, income, and occupational status) and a certain degree of political disillusionment with the country’s main political parties in view of the scarcity of policies to reverse the demographic situation of these areas. As discussed by Southwell et al [51], limited community infrastructure and reduced social cohesion in geographically isolated areas can constrain the diffusion of health information and facilitate the persistence of misinformation, particularly in rural areas such as the empty Spain. This factor may partially explain the observed geographic distribution.

Our findings indicate that individuals with lower socioeconomic status were more likely to be linked to the skeptical group. This aligns with international studies showing that this factor is a clear predictor of health misinformation [52]. However, women were also found to be prone to skepticism, a finding that warrants further investigation to understand gender-specific factors driving COVID-19 misinformation vulnerability, as other studies on gendered scientific misinformation in Spain show that women differentiate misinformative content better than men [49], contrary to the data from our study. It is likely that the role played by misinformation narratives about COVID-19 vaccines and problems with fertility or births is a factor influencing these gender differences [53].

Interestingly, this study highlights the association between institutional trust and misinformed beliefs and conspiracy theories about COVID-19. In the Spanish context, the strong distrust in political parties, the government, and institutions further exacerbated skepticism [38]. While political polarization has been shown to influence misinformation in other countries, our study found that mistrust in health-related institutions, such as the Spanish National Health System and the WHO, was a key determinant of scientifically unsupported opinions linked to skeptical groups in Spain [54]. Therefore, as highlighted in previous studies, this finding underscores the critical role of misinformation in shaping institutional and governmental trust [55], reinforcing the necessity for health institutions to re-establish credibility through transparent communication strategies and consistent messaging. Moreover, as Agley and Xiao [30] point out, it is necessary to keep in mind that believing in false information or conspiracy theories does not necessarily imply that a person cannot simultaneously believe in the official scientific versions. Consequently, simply repeating accepted scientific explanations will not necessarily stop people from believing false information (eg, people who have trust in vaccines but reject COVID-19 vaccines) or even, in practice, combining scientific and anecdotal evidence to make health decisions. Thus, new strategies are needed to strengthen scientific literacy and confidence in science through research transparency and outreach by those individuals working in the scientific field, along with specific knowledge about new technologies that, like the new RNA vaccines, raise more doubts among the population in our countries.

Contrary to some findings that suggest a strong protective role for digital health literacy against misinformation, this study did not find a significant relationship between digital literacy (as measured by the eHealth Literacy Scale) and susceptibility to health misinformation [56]. Instead, the quality of the information sources emerged as a more decisive factor. Individuals who relied on health professionals, institutional websites, and official media (eg, newspapers) were less likely to be misinformed and subsequently skeptical about COVID-19–related topics. These findings suggest that public health interventions should promote reliable and well-identifiable information sources over simply enhancing digital literacy skills. In addition, consistent with global studies, our analysis also found that individuals in the skeptical group were more likely to share health-related information on social media frequently [57]. This behavior can be linked to the psychological need for validation within communities, which also drives the spread of misinformation and the formation of echo chambers in which possibly many of these beliefs are fed back [58]. Thus, public health campaigns should not only aim at promoting critical thinking but also address the emotional and psychosocial aspects of information and misinformation sharing.

A distinctive aspect of this study was the nuanced role of political ideology in misinformation beliefs. Thus, although like other international studies we identified the strong association between ideologically extreme positions and greater susceptibility to misinformation [16], our results suggest that those who did not disclose their political stance were more likely to belong to the skeptical or hesitant groups, a finding that could be linked to the political disaffection that currently exists in Spain among voters on both sides of the political spectrum. This finding, combined with data on the geographic distribution of the hesitant and skeptical groups around the empty Spain, underscores the importance of considering both political disaffection and extremism as key drivers of health misinformation and social polarization in health behaviors in the Spanish context [59].

Finally, in interpreting our findings, it is essential to recognize that not all misinformation operates with equal epistemic weight or public impact. As emphasized in the report by National Academies [27], some claims, such as those concerning vaccine safety, are clearly at odds with established scientific evidence, whereas others may stem from misunderstandings of evolving science or reflect contested interpretations. Therefore, this complexity in apprehending the inherently fluid nature of misinformation highlights the need for nuanced classification and targeted interventions, particularly in public health communication strategies that aim to distinguish deliberate disinformation from more benign forms of confusion or outdated beliefs.

### Limitations

Despite these interesting findings, our work is not without limitations. Given the observational nature of this study, it is not possible to draw causal inferences from the results. Similarly, the cross-sectional design of this study does not allow us to determine the extent to which the responses remain influenced by the social changes brought about by the measures implemented during the pandemic. In addition, the clustering techniques used are not free from potential errors in classifying individuals into groups. Nevertheless, in this study, we have opted to combine the best model fit with criteria of simplicity, interpretability, parsimony, and applicability to other international contexts. In any case, it is also important to highlight the strengths of our work. First, we have identified misinformation topics related to COVID-19 that continue to generate doubts and polarization among the Spanish population. Second, to the best of our knowledge, our study is the first to measure the prevalence of convinced, hesitant, and skeptical groups around COVID-19–related topics in Spain using a nationally representative survey. Third, we have provided a comprehensive characterization of the different social profiles that are susceptible to misinformation in Spain and described their geographic distribution. In summary, our study offers crucial insights into the scope of measures that should be adopted and, particularly, the social determinants that could be targeted to combat misinformation.

### Conclusions

This study provides a comprehensive overview of COVID-19–related misinformation in Spain, offering valuable insights into the social, economic, and ideological factors that influence susceptibility to false or misleading information. By identifying and profiling the convinced, hesitant, and skeptical groups, we have demonstrated the significant polarization surrounding health-related issues, such as vaccine hesitancy about the new vaccines and conspiracy theories. These findings emphasize the importance of targeted interventions to improve public understanding of complex health information to combat the global threat of misinformation. Our findings highlight the critical role of institutional trust in shaping public attitudes and health behaviors, underscoring the need for health and governmental institutions to rebuild credibility through transparent communication and consistent messaging. In addition, the influence of socioeconomic status, political ideology, and information sources on misinformation susceptibility suggests that addressing these structural determinants is essential for the design of effective public health strategies.

From a broader perspective, this study contributes to the global research stream on the impact of misinformation on health behaviors, particularly in the context of future pandemics or health crises. Future research should explore longitudinal approaches to examine how misinformation beliefs evolve over time and assess the effectiveness of interventions designed to reduce the spread of false information. Furthermore, exploring the role of digital literacy, alongside strategies to promote reliable, evidence-based information sources, is crucial to addressing the challenges of emerging information and misinformation ecosystems, especially as we await the full impact of new artificial intelligence technologies. Our findings highlight the necessity of a multifaceted approach that integrates education, health policy development, media platforms, and community engagement to effectively counter the persistent menace of health misinformation.

## Abbreviations

EFA: exploratory factor analysis
WHO: World Health Organization
